# A small number of surgeons outside the control-limit: an observational study based on 9,482 cases and 208 surgeons performing primary total hip arthroplasties in western Sweden

**DOI:** 10.1080/17453674.2020.1772584

**Published:** 2020-06-08

**Authors:** Per Jolbäck, Emma Nauclér, Erik Bülow, Hans Lindahl, Maziar, Mohaddes

**Affiliations:** a Department of Orthopaedics, Institute of Clinical Sciences, The Sahlgrenska Academy, University of Gothenburg, Gothenburg;; b Department of Orthopaedics, Skaraborg Hospital, Lidköping;; c Swedish Hip Arthroplasty Register, Gothenburg;; d Research and Development Centre, Skaraborg Hospital, Skövde, Sweden

## Abstract

Background and purpose — Feedback programs relating to surgeon levels have been introduced in some orthopedic quality registers around the globe. The aim of an established surgeon feedback program is to help surgeons understand their practice and enable an analysis of their own results. There is no surgeon feedback program in Sweden in the orthopedic quality registers and there is a fear that a feedback system might pinpoint surgeons as poor performers, partly due to patient case mix. As a step prior to the introduction of a future possible feedback program in Sweden, we assessed the variation in the occurrence of adverse events (AE) within 90 days and reoperations within 2 years between surgeons in western Sweden and explored the number of surgeons outside the control-limit following primary total hip arthroplasties (THAs).

Patients and methods — Patient data, surgical data, and information on the surgeons, relating to surgeries performed in 2011–2016, were retrieved from 9 publicly funded hospitals in western Sweden. Data from medical hospital records, the Swedish Hip Arthroplasty Register (SHAR) and a regional patient register located in western Sweden were linked to a database. Funnel plots with control-limits based on upper 95% and 99.8% confidence intervals (CI) were used to illustrate the variation between surgeons in terms of the outcome and to explore the number of surgeons outside the control-limit. Both observed and standardized proportions are explored. The definition of surgeons outside the control-limit in the study is a surgeon above the upper 95% CI.

Results — The study comprised 9,482 primary THAs due to osteoarthritis performed by 208 surgeons, where 91% of the included primary THAs were performed by orthopedic specialists and 9% by trainees. The mean overall annual volume for all surgeons was 27. The observed overall mean rate for AEs within 90 days for all surgeons was 6.2% (5.8–6.7) and for reoperations within 2 years 1.8% (1.7–2.2). The proportion of surgeons outside the 95% CI was low for both AEs (0–5%) and reoperations within 2 years (0–1%) in 2011–2016. The corresponding numbers were even lower for AEs (0–3%) but similar for reoperations (0–1%) after standardization for differences in case mix. In a sub-analysis when the number of surgeries performed was restricted to more than 10 primary THAs annually to being evaluated, almost half or more of all the surgeons were excluded from the annual analysis. The result of this restriction was that all surgeons outside the control-limit disappeared after standardization for both AEs and reoperations for all the years investigated. Considering the complete period of 6 years, less than 1% (1 high-volume surgeon for AEs and 2 high-volume surgeons for reoperations) after risk adjustments were outside the 95% CI, and no surgeons were outside the 99.8% CI.

Interpretation — In a Swedish setting, the variation in surgeon performance, as measured by AEs within 90 days and reoperations within 2 years following primary THA, was small and 3% or less of the surgeons were outside the 95% CI for the investigated years after adjustments for case mix. The risk for an individual surgeon to be regarded as having poor performance when creating surgeon-specific feedback in the SHAR is very low when volume and patient risk factors are considered.

In 1975, the 1st orthopedic quality register, the Swedish Knee Arthroplasty Register (Robertsson et al. 2000, Malchau et al. 2018), was started and, 4 years later, it was followed by the Swedish Hip Arthroplasty Register (SHAR) (Kärrholm 2010). These 2 quality registers have played an important role as models for the fair number of successful registers in other countries (Malchau et al. 2018). Today, almost all orthopedic registers publish an annual report with results aggregated at hospital level. Some of the registers have also developed programs for providing surgeon-level feedback and benchmarking data with other surgeons (National Joint Register 2015, Australian Orthopaedic Association National Joint Replacement Registry 2017). The main aim of the feedback programs at surgeon level, hosted by quality registers, is to help surgeons understand their practice.

The models used for visualizing single surgeons and benchmarking between peers in the National Joint Register for England, Wales, Northern Ireland, the Isle of Man and the States of Guernsey (NJR) and the Australian Orthopaedic Association National Joint Replacement Registry (AOANJRR) is funnel plots (Spiegelhalter 2005). The funnel plot has been suggested to be an appropriate statistical technique for reporting surgeon outcomes (Walker et al. 2013). The AOANJRR adjusts surgeon-level data for patients’ age and sex but not for other factors, such as BMI, comorbidities, and smoking, which have been suggested to influence the risk of AE and reoperation following arthroplasty (Mantilla et al. 2002, Thörnqvist et al. 2014, Duchman et al. 2015, Singh et al. 2015, Lübbeke et al. 2016). As yet, none of the Swedish orthopedic registers has started a feedback program to provide individual surgeon data. Little is known about the surgeon performance in a Swedish setting. Here, we describe the variation in outcomes of AE within 90 days and reoperations within 2 years following primary total hip arthroplasties (THAs) among surgeons in western Sweden and explore the number of surgeons outside the control-limit.

## Patients and methods

**Figure 1.**
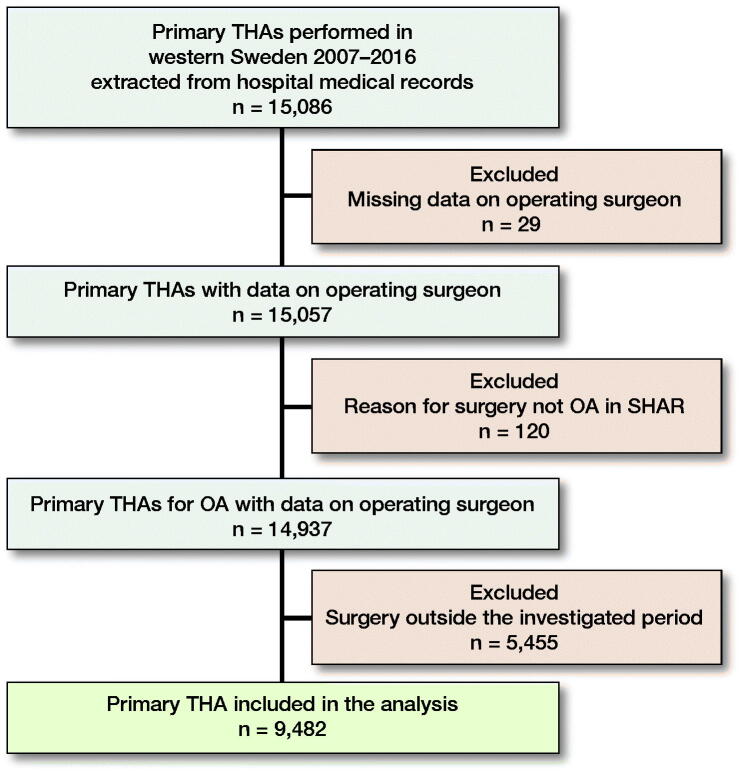
Flow chart.

All primary THAs in patients with a diagnosis of osteoarthritis (OA) of the hip, performed in hospitals managed by the county council of western Sweden between 2011 and 2016, were included in the study (Figure 1). Hospital medical records, the SHAR and the regional patient register, Vega (hereafter only namned regional patient register) were used as data sources. A complete list of sources for the variables that were used, including confounders, is shown in the Supplementary data (Appendix). The link between hospital medical records and the SHAR was made using the 10-digit personal identity number (PIN), the name of the hospital, and the date of surgery. If divergent information was obtained from the SHAR and the hospital medical records, the information in the SHAR was regarded as superior. The linked dataset, containing information from hospital medical records and the SHAR, was subsequently forwarded to the regional patient register to add all AEs and the data were made anonymous by replacing the PIN with a unique identifier.

### Swedish Hip Arthroplasty Register

The aim of the SHAR is to register all primary THAs and reoperations performed in Sweden (Kärrholm 2010). Although participation is voluntary for both hospitals and patients, the completeness and coverage of the SHAR have been high during the past few decades (Kärrholm et al. 2018). The variables recorded in the SHAR include patient factors such as age, sex, diagnosis for implantation, BMI, ASA classification, and technical details on the surgery, such as fixation technique and type of implant.

### Vega­—a regional patient register

The regional patient register was initiated in 2000. It is an aggregated database, containing records relating to all healthcare contacts (both publicly and privately funded) for all the residents in western Sweden. The population in western Sweden was approximately 1.6 million people in 2011 and 1.7 million people in 2016, which constitutes approximately 17% of all the residents in Sweden. The regional patient register provides information to the National Patient Register (NPR) (Ludvigsson et al. 2011). The PIN is used as the unique identifier of all entries in the regional patient register. The regional patient register contains details on the depiction of the caregiver at the point of contact, for example, the level of hospital or elective care, diagnoses, interventions, and length of stay in hospital.

### AEs and reoperations

The definition of AEs we used is the same as in the SHAR and was presented in their 2018 Annual Report (Kärrholm et al. 2018). It has also been used in previous studies (Berg et al. 2018, Jolbäck et al. 2019). The AE code list includes both surgical complications (local complications, secondary fractures, tendon ruptures in the lower extremities), and medical complications (thromboembolic events, myocardial infarction, pneumonia, gastroduodenal ulcers, acute kidney injury, and urinary retention). Reoperations are defined as any further surgery following the index surgery on the previously operated hip. All diagnoses for computing AEs were retrieved from the regional patient register, while the reoperations were retrieved from the SHAR.

### Statistics

Continuous data are presented as means (SD), while categorical data are presented as proportions. Funnel plots were used to visualize variations between surgeons in the proportion of AEs and reoperations respectively and to explore the number of surgeons outside the control-limits. The control-limits are based on upper 95% and 99.8% confidence intervals (CI). Wilson’s method suitable for low n was used to construct the CIs (Brown et al. 2001). The control-limits are dependent on the sample size; a small sample size increases the control-limits and a larger sample size reduces the limits (i.e., surgeons undertaking few surgeries will have a wider control-limit). Both observed and standardized proportions are explored. The standardized proportion was calculated for each surgeon as the ratio of the number of observed events divided by the number of expected events, multiplied by the overall proportion of events (Spiegelhalter 2005). Logistic regression with adjustments for patient risk factors was used to determine the probability of an event for a patient. The expected number of events for a surgeon was estimated by summing the predicted values of an event for the surgeon’s patients. The variables age, sex, ASA classification, BMI, and diagnosis for implantation were assumed to be related to the outcome (Mantilla et al. 2002, Thörnqvist et al. 2014, Duchman et al. 2015, Singh et al. 2015, Lübbeke et al. 2016). For AEs within 90 days, all 5 predictors were included in the logistic regression model. For reoperations within 2 years, the best-performing model included sex, ASA classification, and BMI.

Only cases with complete data were included in the analysis. Patients undergoing simultaneous bilateral primary THAs were included as 1 surgery in the study; 43 patients underwent simultaneous bilateral primary THAs. Staged bilateral primary THAs were performed on 732 patients. We also performed a sub-analysis including surgeons performing more than 10 THAs annually (Walker et al. 2013).

SPSS version 25 (IBM Corp, Armonk, NY, USA) and R version 3.2.3 (R Foundation for Statistical Computing, Vienna, Austria) (R Core Team 2019) were used for the statistical analysis.

### Ethics, funding, and potential conflicts of interest

The study was approved by the Central Ethical Review Board in Stockholm (DNR Ö 9-2016). A research grant for the project was received from Skaraborgs Hospital research foundation. There is no conflict of interest. 

## Results

The analysis included 208 surgeons from 9 public hospitals in western Sweden who performed the 9,482 primary THAs due to OA (Table 1). The categorization of the hospitals included was 1 university-regional hospital, 3 county hospitals, and 5 rural hospitals (based on the SHAR’s categorization of hospitals). Of the 9,482 primary THAs included in the analysis, 8,636 (91%) were performed by orthopedic specialists and 846 (9%) by trainees. The mean annual volume of primary THAs for all surgeons and all years was 27 (SD 17). The mean annual volume of primary THAs varied (Table 2). The annual number of surgeons performing primary THAs decreased in the latter part of the period investigated (Table 3).

**Table 2.** Mean annual surgeon volume during the period investigated

	Annual
	surgeon
	volume
Year	Mean (SD)
2011	21 (12)
2012	24 (16)
2013	24 (14)
2014	31 (19)
2015	28 (16)
2016	34 (17)
All years	27 (17)

**Table 1.** Patient demographics for each year included in the study. Values are number (%) unless otherwise specified

Sex						
	2011	2012	2013	2014	2015	2016
	n = 1,299	n = 1,320	n = 1,569	n = 1,774	n = 1,733	n = 1,787
Age, mean (range)	69 (21–95)	69 (22–93)	69 (20–92)	69 (26–97)	69 (17–97)	68 (21–94)
Male	538 (41)	561 (43)	637 (41)	730 (41)	743 (43)	733 (41)
Female	761 (59)	759 (58)	932 (59)	1,044 (59)	990 (57)	1,054 (59)
BMI, mean (range)	27 (16–49)	27 (15–53)	28 (10–63)	28 (16–61)	28 (15–72)	28 (16–68)
Missing **^a^**	111 (9)	110 (8)	92 (6)	101 (6)	88 (5)	25 (1)
ASA classification						
I	325 (25)	338 (26)	431 (28)	452 (26)	401 (23)	394 (22)
II	745 (57)	766 (58)	903 (58)	1,056 (60)	1,043 (60)	1,083 (61)
III	182 (14)	171 (13)	202 (13)	223 (13)	225 (13)	305 (17)
IV	3 (0.2)	4 (0.3)	3 (0.2)	3 (0.2)	2 (0.1)	1 (0.1)
Missing **^a^**	44 (3)	41 (3)	30 (2)	40 (2)	62 (4)	4 (0.2)
Diagnosis for implantation						
Primary OA	1,226 (94)	1,249 (95)	1,498 (96)	1,681 (95)	1,651 (95)	1,694 (95)
Secondary OA	73 (6)	71 (5)	71 5)	93 (5)	82 (5)	93 (5)
Missing	0 (0)	0 (0)	0 (0)	0 (0)	0 (0)	0 (0)

BMI = body mass index, ASA = American Society of Anesthesiologists, OA = osteoarthritis.

**^a^** 1 hospital (with only 2 surgeons operating during the period) did report a low level of ASA classification and BMI. In 2016 it stopped producing primary THAs. Therefore, there is a large reduction in missing values in 2016 for ASA classification and BMI.

**Table 3.** Annual number of surgeons outside the control-limit (above the upper 95% CI) in funnel plots due to AE within 90 days and reoperations within 2 years for all surgeons regardless of annual surgeon volume of primary THAs

	2011	2012	2013	2014	2015	2016
Factor	n = 116	n = 121	n = 122	n = 116	n = 110	n = 98
Number of surgeons outside the control-limit for adverse events within 90 days						
Observed	5	2	6	3	1	0
Standardized **^a^**	3	1	3	1	1	0
Number of surgeons outside the control-limit for reoperations within 2 years						
Observed	0	0	0	1	1	1
Standardized **^b^**	0	0	0	1	0	1

**^a^**Age, sex, ASA classification, BMI, and diagnosis for implantation

**^b^**Sex, ASA classification, and BMI

ASA = American Society of Anesthesiologists, BMI = body mass index,

CI = confidence interval, THAs = total hip arthroplasties.

The overall mean rate for AEs within 90 days for all surgeons was 6.2% (SD 7.3), with a variation during the years between a minimum of 5.8% (year 2013) and a maximum of 6.7% (year 2011). The corresponding proportion for reoperations within 2 years was 1.7% (2011) to 2.2% (2016), with an overall mean rate of 1.8% (SD 3.9%).

During the years 2011–2016, there were few surgeons outside the upper 95% CI. The year with the highest number of surgeons outside the 95% CI for AEs within 90 days was 2013, there were 6 surgeons outside the control-limit and, after standardization for case mix, only 3 surgeons remained outside the limit. The proportion of surgeons outside the 95% CI during the years investigated varied between 0% and 5%.

The proportions of surgeons outside the 95% CI for reoperations within 2 years were also small, with variations between 0% and 1% annually (min–max) when examining both the observed and standardized proportions.

The result of the sub-analysis, when we included surgeons performing more than 10 primary THAs annually, showed that the surgeons who were outside the control-limit for AEs within 90 days were reduced by more than half (observed) and disappeared when standardization were made for case mix (Table 3). For reoperations within 2 years, all the surgeons outside the control-limit disappeared in the sub-analysis, apart from 1 surgeon in 2016, but, after standardization, this remaining surgeon also disappeared (Table 4).

**Table 4.** Annual number of surgeons outside the control-limit (above the upper 95% CI) in funnel plots due to AE within 90 days and reoperations within 2 years when only surgeons with 10 or more primary THAs annually are included in the analysis

	2011	2012	2013	2014	2015	2016
Factor	n = 52	n = 41	n = 54	n = 54	n = 62	n = 51
Number of surgeons outside the control-limit for adverse events within 90 days						
Observed	1	0	1	1	0	0
Standardized **^a^**	0	0	0	0	0	0
Number of surgeons outside the control-limit for reoperations within 2 years						
Observed	0	0	0	0	0	1
Standardized **^b^**	0	0	0	0	0	0

For footnotes, see Table 3

**Figure 2.**
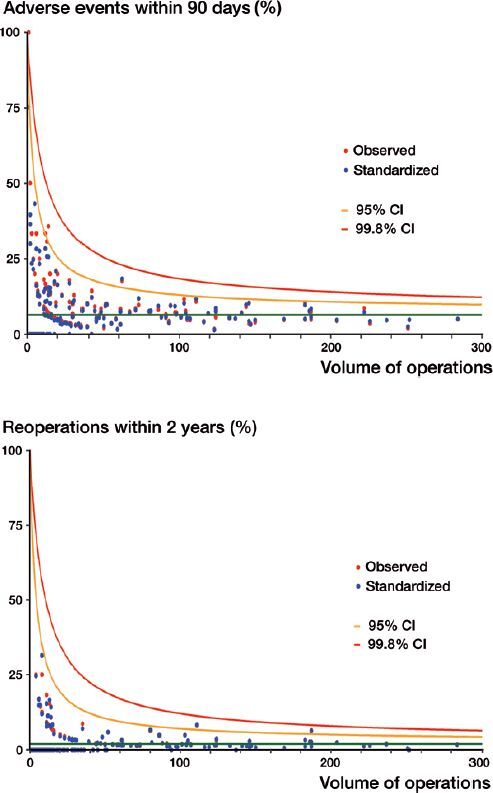
Funnel-plots for AE within 90 days (top panel) and reoperation within 2 years (bottom panel) with the observed and standardized proportions overlaying. The green line is the mean value for the outcome of interest. The yellow line is the 95% CI and the red line is the 99.8% CI. Each dot represents one surgeon. Red dots are the observed proportion and blue dots are the standardized proportion.

Considering the complete period of 6 years, less than 1% (1 high-volume surgeon for AEs and 2 high-volume surgeons for reoperations) after risk adjustments were outside the 95% CI, and no surgeons were outside the 99.8% CI (Figure 2).

## Discussion

Less than 3% of the surgeons were outside the upper 95% CI in this study for both AEs within 90 days and reoperations within 2 years, not only after adjustments for differences in patients’ characteristics but also before any standardization was made. The overall mean rates of both AEs and reoperations in the study are similar to the national average for elective primary THAs in Sweden (Kärrholm et al. 2018).

All the confounders we were able to adjust for are known from earlier studies to influence AEs and the risk of reoperation (Mantilla et al. 2002, Thörnqvist et al. 2014, Duchman et al. 2015, Singh et al. 2015, Lübbeke et al. 2016). However, there could be unknown confounders not available in this study that might affect the outcome following primary THA.

The number of surgeons outside the control-limit due to reoperations within 2 years for patients undergoing surgery between 2015 and 2016 needs to be interpreted with some caution, as the follow-up period for these cases is shorter (0.5–1.5 years) than for the surgeries performed in 2011–2014.

The small number of surgeons outside the control-limit for both AEs and reoperations might be an effect of primary THA surgery being a highly standardized procedure in Sweden, but it might also be an effect of the long tradition of quality registers in Sweden providing feedback at hospital level. Primary THA surgeons in Sweden follow the recommendations given by the SHAR and the relatively large proportion of cemented THAs (Mäkelä et al. 2014, Kärrholm et al. 2017), with a fairly small number of different prostheses accounting for the majority of operations that are reported, may also contribute to the excellent outcomes.

We included only primary THAs due to primary and secondary OA. OA is the most common reason for primary THAs, where four-fifths have OA as a reason for implantation (Kärrholm et al. 2016) during the period of the study. Thus, some of the surgeons included in the study might produce a higher annual volume of primary THAs for reasons other than implantation for OA (e.g., fracture, inflammatory arthritis, femoral head necrosis, childhood disease). The experience from these other primary THAs might contribute to improved outcomes following all THAs for these surgeons and the same improvement in outcomes might be seen between surgeons performing both revision THAs and primary THAs.

The control-limits in our funnel plots are based on CIs, as is the case in the AOANJRR’s feedback system. The AOANJRR has had a lower limit of 50 performed surgeries since the start of the feedback system. We examined the surgeons’ results every year and a fairly large number of surgeons performed only a few operations every year. The number of surgeons outside the control-limit in the study must be interpreted with caution, as we have included all surgeons, regardless of annual surgical volume, and this might increase the uncertainty.

We chose to present individual surgeon variations in funnel plots with control-limits based on CIs using Wilson’s method. The choice of method for constructing control-limits is sensitive when the volume of annual surgeries is low. Wilson’s method was chosen because of the low annual surgeon volume. However, the number of surgeons outside the control-limits should be interpreted with caution when exploring the variation between surgeons with a low annual volume.

When we excluded surgeons performing 10 or fewer primary THAs, almost half or more than half of the surgeons were excluded from the analysis. However, despite this halving of the number of surgeons, we executed the sub-analysis and the findings in this sub-analysis not only halved the number of surgeons, it also reduced or removed the number of surgeons outside the control-limit for both AEs and reoperations. Perhaps there is a “lower volume issue” that needs to be considered in order to make a reliable comparison between surgeons, and not only a problem with the case mix in terms of differences.

Only 9% of the primary THAs was operated on by trainees. This small number of procedures performed by trainees might reflect the trainee education system in Sweden where almost all hospitals educate their own trainees. We can only speculate as to whether trainees are more likely to be outside the control-limit than trained surgeons. The reason for this is that in Sweden a trainee can apply for specialist certification in orthopedics at the Swedish National Board of Health and Welfare after fulfilling the requirements of the orthopedic trainee program at any time of the year. Therefore, a surgeon could have been both a trainee and orthopedic specialist during the same year. However, trainees or newly certified specialists are more probably likely to be low-volume surgeons, and thereby have an increased risk of being regarded as poor performers compared with more experienced surgeons (Ravi et al. 2014, Koltsov et al. 2018).

The small number of surgeons outside the control-limit for both AEs and reoperations in our study might be in conflict with the development of an individual surgeon feedback program following primary THAs in Sweden. However, there might be other aspects and benefits of an individual surgeon feedback program rather than presenting surgeons outside the control-limits, such as general information on individual surgeons’ practice, a substitute for former clinical follow-up visits to the operating surgeon, etc. Further research is needed to explore whether there are other aspects, benefits, or doubts from the surgeons’ point of view on the development of a feedback program.

One strength in this study is that we have been able to adjust for BMI and ASA classification. These 2 confounders are recorded in the SHAR’s standard collection of variables and it is therefore easy to add them to a possible future program for individual surgeon feedback.

One limitation in our study is that only primary THAs performed within the region of western Sweden were included. Some of the surgeons involved in the study might have had temporary or partial employment, having performed primary THAs outside the region investigated. Due to the terms of employment laws in Sweden, it is very uncommon for surgeons to perform surgeries for multiple employers. We anticipated that the limited number of surgeons operating outside the region of western Sweden would not influence our conclusions.

Our study also shares the same limitation as all observational studies using administrative data. Both changes in practice during the study period and local trends, as well as differences in registration, might occur between the included hospitals during the period investigated. The regional patient register we used has not been validated on its own, but it provides data to the NPR. The Swedish National Inpatient Register (IPR) is part of the NPR. The IPR has been validated and contains 99% of all hospital discharges (Ludvigsson et al. 2011). We used a definition of AEs and reoperations requiring hospital admission. We therefore believe that our data are robust and our conclusions are valid.

In summary, the variation in surgeon performance, as measured by AEs within 90 days and reoperations within 2 years following primary THA, was small and 3% or less of the surgeons were outside the 95% control-limit for the years investigated after adjustments for case mix. The risk for an individual surgeon to be regarded as having poor performance when creating surgeon-specific feedback in the SHAR is very low when volume and patient risk factors are considered.

## Supplementary Material

Supplemental Material

## Supplementary data

The Appendix is available as supplementary data in the online version of this article, http://dx.doi.org/10.1080/17453674. 2020.1772584

## References

[CIT0001] Australian Orthopaedic Association National Joint Replacement Registry (AOANJRR). Annual Report; 2017. Available from https://aoanjrr.sahmri.com/annual-reports-2017

[CIT0002] Berg U, Bülow E, Sundberg M, Rolfson O. No increase in readmissions or adverse events after implementation of fast-track program in total hip and knee replacement at 8 Swedish hospitals: an observational before-and-after study of 14,148 total joint replacements 2011–2015. Acta Orthop 2018; 89(5): 522–7. 29985681 10.1080/17453674.2018.1492507 PMC6202734

[CIT0003] Brown D L, Cai T T, DasGupta A. Interval estimation for a binomial proportion. Stat Sci 2001; 16(2): 101–17.

[CIT0004] Duchman K R, Gao Y, Pugely A J, Martin C T, Noiseux N O, Callaghan J J. The effect of smoking on short-term complications following total hip and knee arthroplasty. J Bone Joint Surg Am 201597(13): 1049–58. 26135071 10.2106/JBJS.N.01016

[CIT0005] Jolbäck P, Rolfson O, Cnudde P, Odin D, Malchau H, Lindahl H, Mohaddes M. High annual surgeon volume reduces the risk of adverse events following primary total hip arthroplasty: a registry-based study of 12,100 cases in Western Sweden. Acta Orthop 2019; 90(2): 153–8. 30762459 10.1080/17453674.2018.1554418 PMC6461084

[CIT0006] Koltsov J C B, Marx R G, Bachner E, McLawhorn A S, Lyman S. Risk-based hospital and surgeon-volume categories for total hip arthroplasty. J Bone Joint Surg Am 2018; 100: 1203–8. 30020125 10.2106/JBJS.17.00967

[CIT0007] Kärrholm J. The Swedish Hip Arthroplasty Register (www.shpr.se). Acta Orthop 2010; 81(1): 3–4. 20170435 10.3109/17453671003635918 PMC2856196

[CIT0008] Kärrholm J, Lindahl H, Malchau H, Mohaddes M, Rogmark C, Rolfson O. The Swedish Hip Arthroplasty Register Annual Report 2015; 2016.

[CIT0009] Kärrholm J, Lindahl H, Malchau H, Mohaddes M, Rogmark C, Rolfson O. The Swedish Hip Arthroplasty Register Annual Report 2016; 2017.

[CIT0010] Kärrholm J, Mohaddes M, Odin D, Vinblad J, Rogmark C, Rolfson O. The Swedish Hip Arthroplasty Register Annual Report 2017; 2018.

[CIT0011] Lübbeke A, Zingg M, Vu D, Miozzari H H, Christofilopoulos P, Uckay I, Harbarth S, Hoffmeyer P. Body mass and weight thresholds for increased prosthetic joint infection rates after primary total joint arthroplasty. Acta Orthop 2016; 87(2): 132–8. 26731633 10.3109/17453674.2015.1126157 PMC4812074

[CIT0012] Ludvigsson J F, Andersson E, Ekbom A, Feychting M, Kim J L, Reuterwall C, Heurgren M, Olausson P O. External review and validation of the Swedish national inpatient register. BMC Public Health 2011; 11: 450. 21658213 10.1186/1471-2458-11-450 PMC3142234

[CIT0013] Malchau H, Garellick G, Berry D, Harris W H, Robertson O, Kärrholm J, Lewallen D, Bragdon C R, Lidgren L, Herberts P. Arthroplasty implant registries over the past five decades: development, current, and future impact. J Orthop Res 2018; 36(9): 2319–30. 29663575 10.1002/jor.24014

[CIT0014] Mantilla C B, Horlocker T T, Schroeder D R, Berry D J, Brown D L. Frequency of myocardial infarction, pulmonary embolism, deep venous thrombosis, and death following primary hip or knee arthroplasty. Anesthesiology 2002; 96(5): 1140–6. 11981154 10.1097/00000542-200205000-00017

[CIT0015] Mäkelä K T, Matilainen M, Pulkkinen P, Fenstad A M, Havelin L I, Engesaeter L, Furnes O, Overgaard S, Pedersen A B, Kärrholm J, Malchau H, Garellick G, Ranstam J, Eskelinen A. Countrywise results of total hip replacement: an analysis of 438,733 hips based on the Nordic Arthroplasty Register Association database. Acta Orthop 2014; 85(2): 107–16. 24650019 10.3109/17453674.2014.893498 PMC3967250

[CIT0016] National Joint Register (NJR). NJR Clinician Feedback User Guide; 2015.

[CIT0017] Ravi B, Jenkinson R, Austin P C, Croxford R, Wasserstein D, Escott B, Paterson J M, Kreder H, Hawker G A. Relation between surgeon volume and risk of complications after total hip arthroplasty: propensity score matched cohort study. BMJ 2014; 348: g3284. 24859902 10.1136/bmj.g3284 PMC4032026

[CIT0018] R Core Team. R: A language and environment for statistical computing. Vienna, Austria: R Foundation for Statistical Computing; 2019. Available from https://www.R-project.org/.

[CIT0019] Robertsson O, Lewold S, Knutson K, Lidgren L. The Swedish Knee Arthroplasty Project. Acta Orthop Scand 2000; 71(1): 7–18. 10743986 10.1080/00016470052943829

[CIT0020] Singh J A, Schleck C, Harmsen W S, Jacob A K, Warner D O, Lewallen D G. Current tobacco use is associated with higher rates of implant revision and deep infection after total hip or knee arthroplasty: a prospective cohort study. BMC Med 2015; 13: 283. 26586019 10.1186/s12916-015-0523-0 PMC4653911

[CIT0021] Spiegelhalter D J. Funnel plots for comparing institutional performance. Stat Med 2005; 24(8): 1185–202. 15568194 10.1002/sim.1970

[CIT0022] Thörnqvist C, Gislason G H, Kober L, Jensen P F, Torp-Pedersen C, Andersson C. Body mass index and risk of perioperative cardiovascular adverse events and mortality in 34,744 Danish patients undergoing hip or knee replacement. Acta Orthop 2014; 85(5): 456–62. 24954493 10.3109/17453674.2014.934184 PMC4164861

[CIT0023] Walker K, Neuburger J, Groene O, Cromwell D, van der Meulen J. Public reporting of surgeon outcomes: low numbers of procedures lead to false complacency. Lancet 2013; 382: 1674.77. 23831144 10.1016/S0140-6736(13)61491-9

