# Supplementary material for: A small number of surgeons outside the control-limit: an observational study based on 9,482 cases and 208 surgeons performing primary total hip arthroplasties in western Sweden
Source: Acta Orthop. 2020 Jun 8;91(5):581–6. doi: 10.1080/17453674.2020.1772584 (PMC8025681; doi:10.1080/17453674.2020.1772584)
Supplement: Supplemental Material [file IORT_A_1772584_SM0118.pdf]

Supplementary data

Appendix. Sources of variables

Factor	Hospital medical records	SHAR	Regional patient register (Vega)
10-digit PIN	X	X	X
Age	X	X	X
Sex	X	X	
ASA classification	X	X	
BMI		X	
Date for surgery	X	X	X
Hospital	X	X	
Name of the surgeon	X		
Diagnosis for implantation	X	X	
ICD-10 codes for AE within 90 days			X
Reoperations within 2 years		X	X

PIN = personal identification number, AE = adverse events, ICD = International Classification of Diseases.
